# Selective Exoenzymatic Labeling of Lipooligosaccharides of *Neisseria gonorrhoeae* with α2,6‐Sialoside Analogues

**DOI:** 10.1002/cbic.202200340

**Published:** 2022-08-23

**Authors:** Hanna de Jong, Maria J. Moure, Jet E. M. Hartman, Gerlof P. Bosman, Jun Yang Ong, Bart W. Bardoel, Geert‐Jan Boons, Marc M. S. M. Wösten, Tom Wennekes

**Affiliations:** ^1^ Department of Chemical Biology and Drug Discovery Utrecht Institute for Pharmaceutical Sciences and Bijvoet Center for Biomedical Research Utrecht University Universiteitsweg 99 3584 CG Utrecht The Netherlands; ^2^ Department of Biomolecular Health Sciences Utrecht University Yalelaan 1 3584 CL Utrecht The Netherlands; ^3^ Complex Carbohydrate Research Center and Department of Chemistry University of Georgia 315 Riverbend Road Athens GA 30602 USA; ^4^ Department of Medical Microbiology University Medical Center Utrecht Heidelberglaan 100 HP G04.614 3584 CX Utrecht The Netherlands; ^5^ Chemical Glycobiology Lab, CIC bioGUNE Basque Research & Technology Alliance (BRTA) Bizkaia Technology Park, Building 800 48160 Derio Spain

**Keywords:** glycobiology, glycoengineering, lipooligosaccharides, sialic acid

## Abstract

The interactions between bacteria and their host often rely on recognition processes that involve host or bacterial glycans. Glycoengineering techniques make it possible to modify and study the glycans on the host's eukaryotic cells, but only a few are available for the study of bacterial glycans. Here, we have adapted selective exoenzymatic labeling (SEEL), a chemical reporter strategy, to label the lipooligosaccharides of the bacterial pathogen *Neisseria gonorrhoeae*, using the recombinant glycosyltransferase ST6Gal1, and three synthetic CMP‐sialic acid derivatives. We show that SEEL treatment does not affect cell viability and can introduce an α2,6‐linked sialic acid with a reporter group on the lipooligosaccharides by Western blot, flow cytometry and fluorescent microscopy. This new bacterial glycoengineering technique allows for the precise modification, here with α2,6‐sialoside derivatives, and direct detection of specific surface glycans on live bacteria, which will aid in further unravelling the precise biological functions of bacterial glycans.

## Graphical abstract

**Selective exoenzymatic labeling** (SEEL) can be applied as a glycoengineering technique for the modification of cell surface glycans on live bacteria; in this case the recombinant glycosyltransferase ST6Gal1 installed synthetic α2,6‐sialoside derivatives with a reporter group on the lipooligosaccharides of the bacterial pathogen *Neisseria gonorrhoeae*.

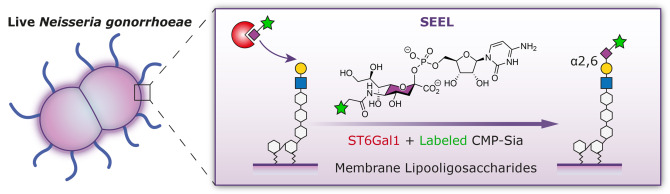

## Introduction

Glycans play a crucial role in many biological processes.^[1]^ They are particularly prevalent on the outside of the cell in the so‐called glycocalyx. Efforts to manipulate and track glycans in the glycocalyx with the use of glycoengineering techniques are gaining momentum.[^2^, ^3^] In glycoengineering, the glycans on a cell surface are modified by either inserting whole glycoconjugates or editing the existing glycan structures by introducing, removing or altering specific monosaccharide residues, which often entails the introduction of a chemical reporter. This approach allows studying specific glycans and the precise modification of their structure in the relevant biological context of a living cell. So far, many of the reported glycoengineering techniques have focused on mammalian cells and have contributed to a better understanding of the structure‐function relationship of glycans within the context of the glycocalyx. However, in case of host‐microbe interactions, comprehending cell surface glycosylation and their biological functions extends not only to mammalian cells, but also to microbial glycans, as illustrated by the many glycan interactions between a host and microbe that influence processes like bacterial pathogenesis, interactions with host receptors, and sialylation to evade immune detection by mimicry of host glycans.[^4^, ^5^]

Currently, few glycoengineering techniques have been reported that can modify bacterial glycans. The most widely applied approach is metabolic oligosaccharide engineering (MOE),[^6^, ^7^] which makes use of the cell‘s own metabolic pathways to incorporate monosaccharides with a chemical reporter group into sugar nucleotides that are eventually incorporated into bacterial glycans by native glycosyltransferases. MOE has been applied on microbes to engineer their cell wall,[^8^, ^9^, ^10^, ^11^, ^12^, ^13^, ^14^, ^15^] to image them,[^16^, ^17^] discover glycoproteins[^18^, ^19^] or to develop new antibacterial strategies.[^20^, ^21^, ^22^] MOE is a powerful approach to selectively label unique bacterial monosaccharides like 3‐deoxy‐d‐manno‐oct‐2‐ulosonic acid (Kdo) in LPS^[23]^ and *N*‐acetyl muramic acid (NAM) in peptidoglycan,[^24^, ^25^] among other residues.[^6^, ^22^, ^26^] Several reports have described MOE with sialic acids, for instance neuraminic acid derivatives for *Haemophilus ducreyi* ^[27]^ or non‐typeable *Haemophilus influenzae*,^[28]^ or legionaminic^[29]^ and pseudaminic^[12]^ acid for the flagella of *Campylobacter jejuni*. However, MOE is not generally applicable to bacteria if the biosynthetic machinery for the metabolic processing of the monosaccharide is absent. Other examples of glycoengineering techniques for bacteria are the chemoenzymatic synthesis of a heptasaccharide of *C. jejuni* ^[30]^ and a modification of a mycobacterial cell wall with a glycolipid derivative.[^31^, ^32^] These pioneering examples of bacterial glycoengineering show the promise of this approach, but current techniques have limited control over the type of glycoconjugate that is modified. They also do not address potential drawbacks of acyl esters that are often used to enhance passive uptake of the probe in MOE.[^33^, ^34^] As most bacteria lack the required esterase activity to remove these acyl esters,[^35^, ^36^] they therefore often require high and potentially cytotoxic extracellular concentrations of the unprotected monosaccharide probe.

Our goal was to investigate whether a glycoengineering technique for mammalian cells, selective exoenzymatic labeling (SEEL)[^37^, ^38^] could be adapted to bacteria and thus expand the glycoengineering toolbox for bacterial glycans to be able to manipulate and study specific glycans on the surface of live bacteria. SEEL uses an exogenously applied recombinant glycosyltransferase to selectively label the glycocalyx of a cell with tailor‐made sugar nucleotide analogs. In earlier work, we reported a sialyltransferase ST6Gal1 that selectively modifies a terminal *N*‐acetyllactosamine (LacNAc) on various human cell lines with α2,6‐sialosides. In a two‐step or one‐step SEEL approach, respectively, the sialic acid either contained a bio‐orthogonal azide that could be clicked to a biotin reporter group, or was already covalently coupled to a biotin.[^37^, ^38^] SEEL is complementary to MOE due to its two unique features. The first one is the ability to precisely modify a particular glycan acceptor site with a specific linkage type because an enzyme of choice can be employed. Second, the monosaccharide derivative does not have to go through the multistep metabolic route of the cell, which allows for the one‐step introduction of more diverse labels, such as a biotin or fluorophore. Considering these strengths of SEEL, we wanted to evaluate if SEEL can be applied to bacteria and thus function as a new strategy to modify their cell surface glycans.

**Figure 1:**
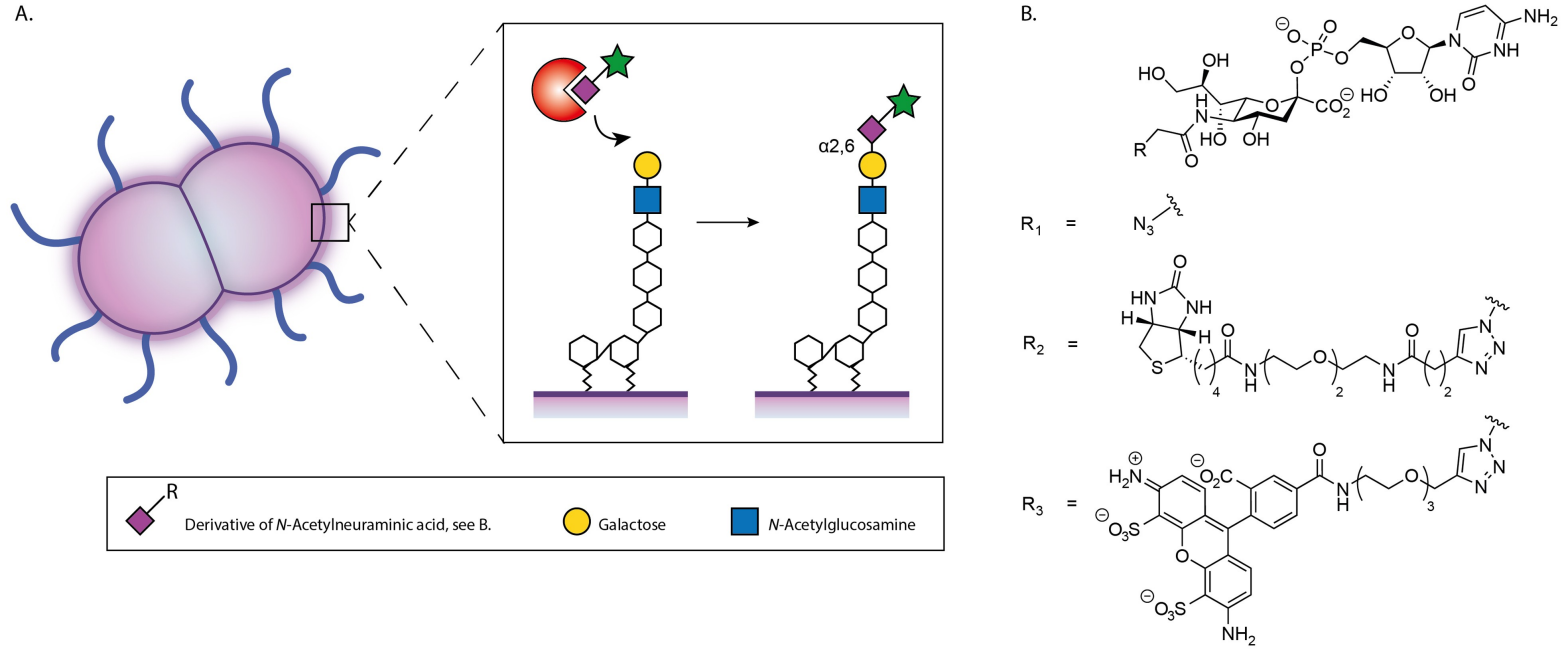
A) Schematic overview of selective exoenzymatic labeling of the lipooligosaccharides on *N. gonorrhoeae* with a sialic acid that contains a reporter group. B) Structures of azido, biotinylated and fluorescent CMP‐Sia derivatives.

We selected *Neisseria gonorrhoeae* as our target, which is a Gram‐negative bacterial pathogen causing the sexually transmitted disease gonorrhea.^[39]^ Its lipooligosaccharides (LOS) contain a terminal *N‐*acetyllactosamine, which can be α2,3‐sialylated by its native sialyltransferases that scavenge CMP‐sialic acid from the host.[^39^, ^40^] The resulting sialylated terminal glycans on the bacterium resemble mammalian *N‐*glycans and glycosphingolipids, and are thus a form of glycan mimicry that conceals the bacteria from immune detection.^[5]^ The use of terminal sialic acids to display glycans that mimic the host extends to other pathogenic bacteria and raises questions about the role of this monosaccharide in the interaction with the immune system, making sialic acid on bacteria a prime target for glycoengineering.^[5]^ Since the established SEEL approach that we developed for mammalian cells uses the recombinant sialyltransferase, ST6Gal1, to modify terminal *N‐*acetyllactosamines of *N*‐glycans with sialic acid derivatives, we hypothesized that *N. gonorrhoeae* would be an ideal candidate for adapting this glycoengineering technique to bacteria (Figure 1).

Here, we report for the first time the successful use of selective exoenzymatic labeling on live bacteria. We show that SEEL labels the LOS of *N. gonorrhoeae* with a preference for LacNAc as the terminal glycan unit and that the introduced α2,6‐linked sialic acid derivatives can be visualized and quantified on the bacteria.

## Results and Discussion

### Glycosyltransferase ST6Gal1 sialylates terminal galactosides on *N. gonorrhoeae* LOS

**Figure 2:**
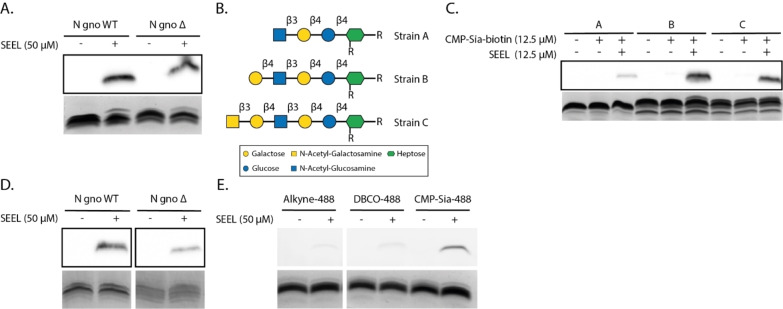
Western blot and silver stain analysis of heat‐inactivated *N. gonorrhoeae* LOS labeled with SEEL. A) In a one‐step reaction, the CMP‐Sia‐biotin is incorporated in the LOS of a wildtype and sialyltransferase mutant *N. gonorrhoeae* (N gno WT and N gno Δ, respectively), as can be seen by the signal on Western blot (upper panel) and the small increase in molecular weight visualized by silver stain (bottom panel). Estimation of the amount of labeling of the LOS by SEEL based on the shift in the silver stained gel can be found in the SI (SI Figure S3). B) Three different strains of LOS on *N. gonorrhoeae* to test labeling specificity. C) Bacteria were treated with only CMP‐Sia‐biotin as a control or SEEL and the LOS was analyzed with Western blot and silver stain. D) In a two‐step reaction, the CMP‐Sia‐azide is incorporated in the LOS of a wildtype and sialyltransferase mutant *N. gonorrhoeae* and then clicked with an alkyne‐biotin. The LOS was analyzed with Western blot and silver stain (raw data images available in the SI). E) In either a two‐step reaction, for alkyne‐488 and DBCO‐488, or a one‐step reaction, with CMP‐Sia‐488, the mutant *N. gonorrhoeae* were labeled. The LOS was analyzed with in‐gel fluorescence (upper panel) or silver stain (bottom panel). The results of this gel are combined in this figure and the raw data images are available in the SI.

We set out to test the glycoengineering technique SEEL on the bacteria *Neisseria gonorrhoeae*. We started by testing one‐step SEEL, using biotinylated cytidine monophosphate sialic acid (CMP‐Sia‐biotin), on two different strains of *N. gonorrhoeae*: one wildtype and one mutant lacking its own sialyltransferase (Δ, ST mutant).[^41^, ^42^] It has been reported that *N. gonorrhoeae* can scavenge cytidine monophosphate *N*‐acetylneuraminic acid (CMP‐Neu5Ac) from the environment.[^43^, ^44^] In addition, it has been shown that the sialyltransferases of *N. gonorrhoeae* can also use azido or microbial sialic acid nucleotide sugars as substrates[^45^, ^46^] to typically install α2,3‐sialosides on its LOS.[^39^, ^47^, ^48^] In order to test the labeling via SEEL and thus to exclude the contribution of these native enzymes in the experiments (SI Figure S1), we either used a sialyltransferase mutant (Δ, ST mutant) or heated the bacteria to inactivate these enzymes and then performed SEEL. For both wildtype and mutant strains, we observed labeling of the LOS on Western blot and on polyacrylamide gel after silver staining through upward shifted bands of the LOS (Figure 2 A). To check for any labeling of other glycoconjugates besides LOS, (glyco)protein samples of SEEL‐treated bacteria were analyzed and labeling of bacterial glycoproteins was not observed (SI Figure S2A). However, the analysis of glycoproteins identified a band that corresponds to the labeled recombinant sialyltransferase ST6Gal1 (47 kDa) that can label its *N*‐glycans during SEEL, as previously observed with mass spectrometry analysis by our group (unpublished data; manuscript in preparation) (SI Figure S2B). From these initial experiments, we could conclude that SEEL is indeed able to selectively label the LOS of *N. gonorrhoeae*.

Next, we wanted to evaluate if SEEL selectively labels *N*‐acetyllactosamine on the terminal position of the LOS. To this end, we labeled three different isogenic *N. gonorrhoeae* strains with different terminal glycan structures, strain B contains LOS with a terminal *N*‐acetyllactosamine, strain A lacks a terminal galactose thus exposing a terminal *N*‐acetylglucosamine and strain C has an additional *N*‐acetylgalactosamine (Figure 2B).^[49]^ After heat treatment and SEEL labeling, it was possible to compare the labeling patterns for the different strains. As expected, weak labeling of strain A was observed and strong labeling of B, but also strong labeling of the LOS of strain C. We assume that strain C is labeled because the enzyme that installs the terminal *N*‐acetylgalactosamine is under phase‐variable expression[^50^, ^51^] and it might thus not be present under the growth conditions, which exposes a terminal *N*‐acetyllactosamine in strain C and makes it closely resemble strain B. A minor amount of labeling was observed for strain A, which we speculate originates from off‐target labeling by ST6Gal1 of a lactose epitope, which is a glycan structure present closer to the core of the LOS of this strain.[^52^, ^53^] This lactose epitope may be accessible for enzymatic modification since *N. gonorrhoeae* does not have capsular polysaccharides and antibodies have also been reported to be able to bind this lactose epitope of the LOS[^54^, ^55^] suggesting accessibility for enzymes as well. Additionally, it has been reported that lactose can be sialylated as well by ST6Gal1, yet the acceptor binding for *N*‐acetyllactosamine is much greater, a 80‐fold increase in Km for lactose compared to *N*‐acetyllactosamine.[^56^, ^57^, ^58^] Taken together, we conclude that when SEEL is applied on *N. gonorrhoeae* with recombinant ST6Gal1 it preferentially labels the LOS terminal *N*‐acetyllactosamines.

### Fluorescently tagged sialosides on *N. gonorrhoeae* LOS allow for quantification of sialylation via ST6Gal1

**Scheme 1:**
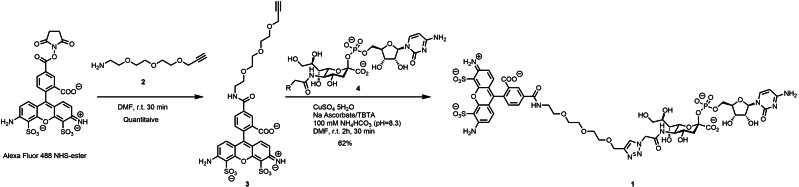
Synthesis of Alexa Fluor 488 CMP‐sialic acid (CMP‐Sia‐AF488). Alexa Fluor NHS was coupled to pegylated alkyne 2, resulting in compound 3. Through a copper‐catalyzed azide‐alkyne cycloaddition (CuAAC) compound 3 was coupled to 4 (CMP‐NeuAz^[38]^), resulting in compound 1, CMP‐Sia‐AF488.

After studying the targeted acceptor glycan of SEEL in *N. gonorrhoeae*, we focused on quantifying the number of bacteria that are being labeled. To achieve this, a fluorescent reporter group was introduced on the bacteria for analysis by flow cytometry. First, we tested the efficiency of two‐step SEEL to introduce a reporter group via a click reaction of the sialosides with an azide to a terminal alkyne connected to a biotin reporter. The copper‐catalyzed azide‐alkyne cycloaddition (CuAAC) resulted in a clear signal (Figure 2D). Next, we evaluated this again, but now for both a CuAAC and strain‐promoted azide‐alkyne cycloaddition (SPAAC) with a terminal alkyne or dibenzocyclooctyne (DBCO) group containing an Alexa Fluor 488 fluorescent dye (AF488). Both these experiments produced only a small amount of fluorescently labeled LOS on gel (Figure 2E). To compare the two‐step SEEL with one‐step SEEL, a CMP‐sialic acid derivative with an AF488 dye, CMP‐Sia‐AF488, was synthesized by conjugating the fluorescent dye to CMP‐NeuAz via a CuAAC reaction (Scheme 1).

**Figure 3:**
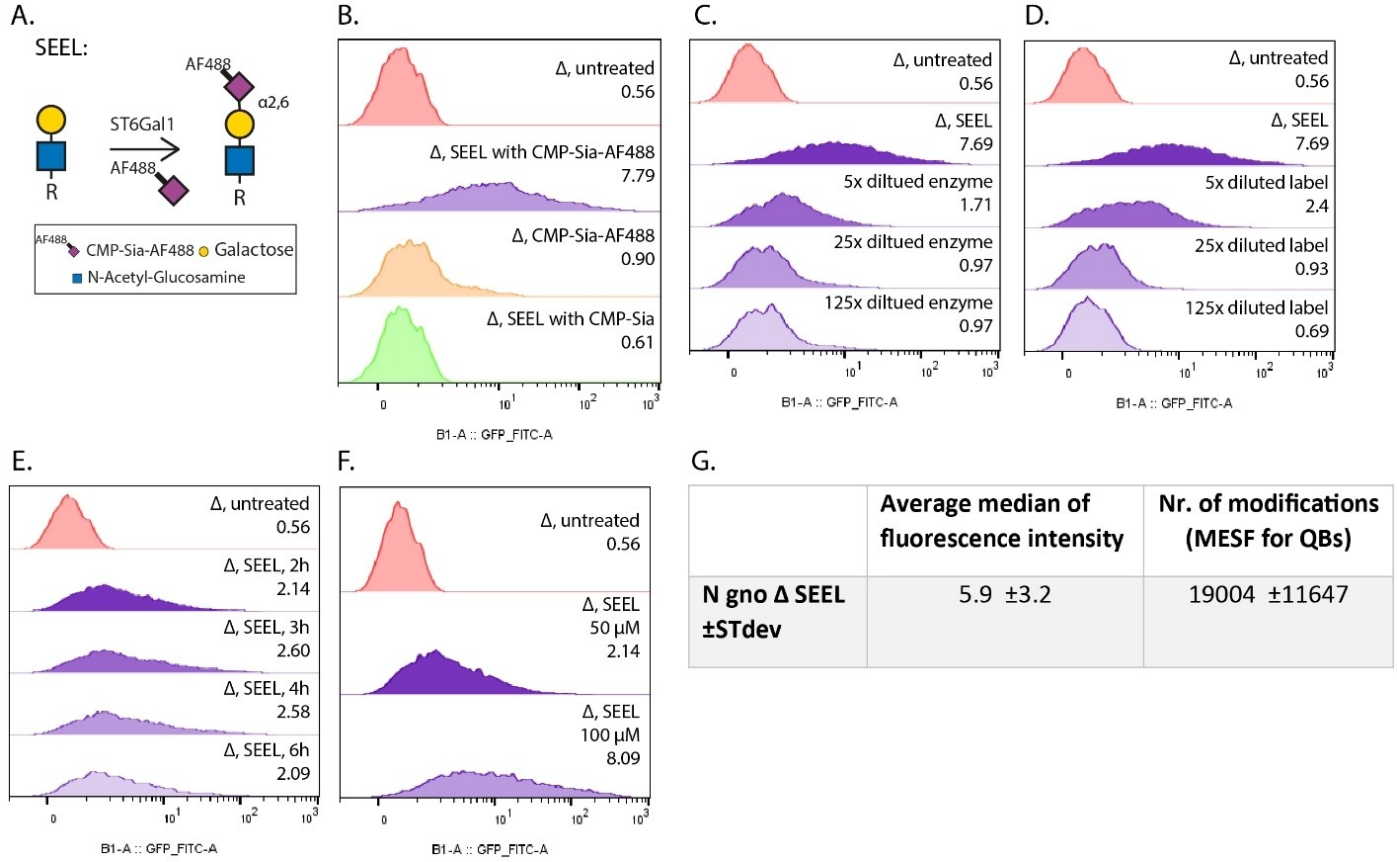
Flow cytometry data to quantify labeling and to test parameters of SEEL treated *N. gonorrhoeae*. A) Schematic overview of the LOS labeling for SEEL treated mutant bacteria. Only the terminal glycans are depicted in this figure for clarity, but the experiments concern whole *N. gonorrhoeae* which were SEEL‐treated. B) Different conditions to confirm that the signal from the SEEL treated label originates from the SEEL treatment after 2‐hour incubation. The median fluorescence intensity is given per condition on the right of each panel. C) Dilution series of the amount of enzyme in the SEEL label mix, 2‐h incubation; decreasing amounts of ST6Gal1 (μg): 1.05; 0.21; 0.042; 0.0084. D) Dilution series of the amount of CMP‐Sia‐AF488 in the SEEL label mix, 2‐h incubation; decreasing concentration CMP‐Sia‐AF488 (μM): 50; 10; 2; 0.4. E) SEEL labeling of *N. gonorrhoeae* after different incubation times. F) SEEL labeling with increased concentration of SEEL label mix. G) Quantification of the amount of fluorescence on SEEL treated bacteria (N gno Δ SEEL) from three independent measurements and the standard deviation (±STdev). The median fluorescent intensity was determined for mutant bacteria labeled by SEEL. This number was converted to the number of modifications (Nr. of modifications) on the cell surface through the Molecules of Equivalent Soluble Fluorochrome (MESF) of the Quantum Beads (QBs).

Compared to the click reaction in the two‐step approach, this fluorescently labeled sugar nucleotide would avoid background labeling often observed with a fluorescent dye interacting with cell surfaces^[59]^ or the potential cytotoxicity of CuAAC.^[60]^ When comparing two‐step and one‐step SEEL we saw a significantly stronger signal for one‐step SEEL, especially for the in‐gel fluorescence (Figure 2E). In previous studies, we made a similar observation while comparing the amount of labeled mammalian glycoproteins with one‐step and two‐step SEEL.^[38]^ We speculate that the two‐step SEEL is less efficient because the click reaction might be sterically hindered on the surface of the bacteria. Since one‐step SEEL would give a more accurate number of labeled bacteria, we continued using one‐step SEEL with our newly synthesized CMP‐Sia‐AF488 for flow cytometry experiments. These measurements on the SEEL treated *N. gonorrhoeae* ST mutant demonstrated that there is an increase in fluorescence compared to the controls (Figure 3B) in which either only CMP‐Sia‐AF488 was added, or SEEL treated bacteria with natural CMP‐Neu5Ac. Although the flow cytometry data showed that most mutant bacteria were being labeled with SEEL (Figure 3B), it appears as a broad distribution. In attempt to further optimize the SEEL protocol, we varied several parameters that showed the labeling could be increased with higher concentrations of label mix, but longer incubations times showed similar labeling as 2 hour incubation, and less enzyme or nucleotide sugar even showed a decrease in labeling (Figure 3C–F). Next, we were interested in gaining insight into the amount of sialic acid incorporated on a bacterium's surface by SEEL. To determine the median number of fluorescently labeled sialosides per bacteria by SEEL, we used quantum beads with a known number of fluorophores to make a calibration curve (SI Figure S4).^[61]^ This revealed that SEEL treated bacteria on average have 19000 modifications with fluorescently modified sialic acid on their LOS (Figure 3G). Taken together with the flow cytometry data, which showed a broad distribution of fluorescence intensity that partially spanned into the same intensity as for the unlabeled bacteria, this shows that most of the SEEL treated bacteria are labeled, but that labeling of the LOS is not complete. This can also be deduced from the increased level of sialylation that is observed on gel (SI Figure S1) when SEEL is performed with ST6Gal1 in the presence of native α2,3‐sialyltransferases, and in flow cytometry (SI Figure S5). This indicates that the native sialyltransferases are able to access more acceptor sites, but this is not useful for the application of SEEL as it leads to a mixture of α2,6 and 2,3‐sialosides. Additionally, the LOS bands that have not completely shifted after SEEL labeling (Figure 2A) indicate that labeling of the LOS is not complete and a higher number of modifications might be achieved.

### Fluorescently tagged sialosides introduced by SEEL on live *N. gonorrhoeae* allows for direct imaging of LOS

**Figure 4:**
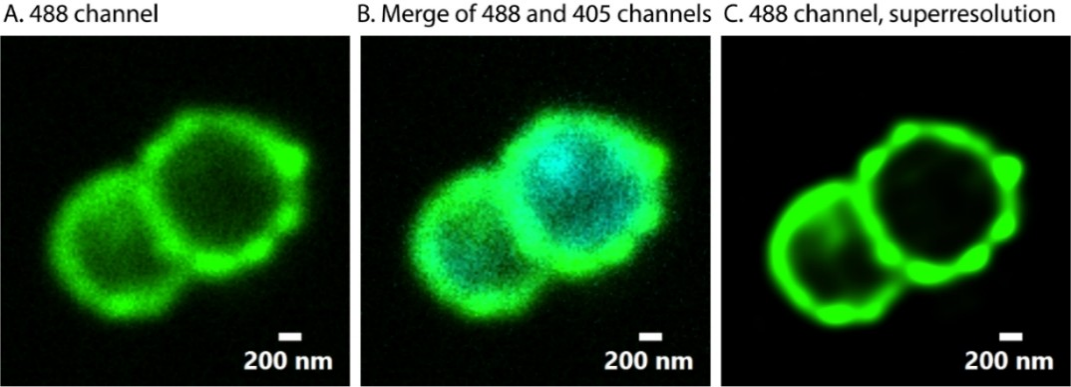
Fluorescence microscopy images show that *N. gonorrhoeae* are fluorescently labeled on the outside of the cell. A) 488 channel; B) merge of 488 and 405 (DAPI) channels; C) 488 channel with super resolution.

Finally, we wanted to visualize the SEEL introduced sialic acid analogue. Through SEEL it is possible to label intact bacteria and since the enzyme ST6Gal1 is exogeneous, labeling takes place extracellularly.^[62]^ The aim was to visualize the fluorescent reporter group on the bacteria that was introduced by SEEL. Fluorescence microscopy of the *N. gonorrhoeae* ST mutant that was SEEL treated with CMP‐Sia‐AF488 showed a bright green signal in a circular shape around the cytoplasm in which the chromosomal DNA was stained with DAPI, and thus indicating successful labeling with CMP‐Sia‐AF488 (Figure 4). In agreement with the flow cytometry data, fluorescence microscopy showed a similar ratio of fluorescently labeled versus unlabeled *N. gonorrhoeae* by SEEL (SI Figures S6 and S7).

### SEEL in comparison with other glycoengineering techniques

These combined results demonstrated that it is possible to adapt SEEL to bacteria. The power of SEEL, as a glycoengineering technique, is that it is targeted because it incorporates one type of monosaccharide on a specific acceptor on the cell surface, in this case Neu5Ac on the terminal *N*‐acetyllactosamine of LOS. Additionally, the use of an exogenous enzyme ensures that a certain linkage type between the glycans is made because of the inherent specificity of the chosen glycosyltransferase, which can be a glycosidic linkage of choice that is non‐native for the bacteria, and that the modification is presumably made extracellularly. In case of *N. gonorrhoeae*, SEEL can introduce an α2,6‐ glycosidic linkage on strains that often have α2,3‐linked sialic acid.[^39^, ^47^, ^48^, ^63^] This same non‐native glycosidic linkage was introduced on isolated Neisserial LOS by Mandrell et al.^[64]^ with an exogeneous sialyltransferase and radiolabeled sialoside, CMP‐[^14^C]‐Neu5Ac. In that study, it was estimated that less than ten percent of the radiolabeled sialoside was transferred to the isolated LOS. In comparison, we report here for the first time a non‐native glycosidic linkage on live bacteria and with CMP‐Sia derivatives containing chemical reporter groups such as an azide, biotin or fluorescent dye. An additional advantage of SEEL, is that it can introduce an extracellular modification in a single step; it has the potential to introduce specific cell surface modifications with large biomolecules.^[65]^ The single step introduction of a fluorescently labeled sialoside, as shown here, is for instance unlikely via MOE with a monosaccharide derivative because the metabolic enzymes would need to accept the fluorophore derivative in multiple steps.^[66]^ Currently, MOE is the most widely applied approach to glycoengineer bacterial glycans. Although this technique has the power to hijack the metabolic process of the cell to incorporate unnatural glycans, it has some disadvantages due to this requirement of metabolic processing of the externally added monosaccharide derivatives.^[35]^ A case in point is that MOE with a monosaccharide derivative cannot be applied to *N. gonorrhoeae* to engineer sialic acid (SI Figure S8), because the monosaccharide derivative cannot be converted to the nucleotide sugar since the bacteria lacks the required CMP synthetase in the metabolic pathway.^[39]^ Another advantage of SEEL is that it did not show cell toxicity for *N. gonorrhoeae* (SI Figure S9). A possible limitation of SEEL includes the number of modifications made (Figure 3). The origin of the broad distribution of SEEL labeled bacteria we observed in flow cytometry poses an in interesting subject for follow‐up research and might be attributed to different growth phases and thus different cell surface architectures of bacteria, the arrangements of the cocci, or the local concentration of the exogeneous enzyme. On the other hand, SEEL has the unique property of being able to install a sialoside with a linkage of choice, here a non‐native α2,6‐linkage. This type of glycosidic linkage cannot be obtained through the bacterial biosynthetic machinery during MOE or labeling through native sialyltransferases.[^43^, ^44^, ^45^, ^46^] The reported activity of these native bacterial enzymes could also mean that knockouts of these are required to exclude their contribution for certain applications, like the introduction of sialic acids on specific acceptor glycans to study specific biological processes.

## Conclusion

In conclusion, here we show for the first time that SEEL can also be applied as a glycoengineering technique for the modification of cell surface glycans on live bacteria, in this case synthetic α2,6‐sialoside derivatives with a reporter group on the LOS of *N. gonorrhoeae*. Bacterial SEEL represents a promising new complementary technique to engineer microbial glycans, next to MOE, with the advantage that SEEL can introduce a (non‐native) glycosidic linkage and terminal monosaccharide derivative of choice by selecting or developing the suitable recombinant glycosyltransferase.

In the future, the scope of SEEL to label other bacteria with a terminal LacNAc like *N. meningitidis* or *H. ducreyi* will be explored, as well as the use of this technique to study the interactions between sialylated LOS of *N. gonorrhoeae* and its host. In addition, the principle behind SEEL can be expanded to other glycans of interest, with the corresponding sugar nucleotide derivatives and glycosyltransferases, to study their role on the LOS/LPS during host‐microbe interactions.

## Experimental Section

### Materials

α‐(2,6)‐Sialyltransferase (ST6Gal1), CMP−Sia−N_3_ and CMP−Sia‐biotin were prepared as reported.^[38]^ Alkaline phosphatase (FastAP) was purchased from Thermo Fisher Scientific (EF0651). HRP conjugated anti‐biotin antibody (200‐032‐211) was purchased from Jackson ImmunoResearch Laboratories. Acetylene‐PEG_4_‐biotin (CLK‐TA105), DBCO‐PEG_4_‐biotin (CLK‐A105P4), AF488‐alkyne (CLK‐1277), and DBCO‐AF488 (CLK‐1278) were purchased from Jena Bioscience.

Chocolate Columbia agar plates were purchased from BioTrading (K018P090KP). Peptone was purchased from Oxoid (LP0085).

### Bacterial strains and culture

*N. gonorrhoeae* F62 WT, Δ STase, isogenic strains A, B and C, were gifted by Prof. Dr. Jos van Putten (Utrecht University).[^41^, ^42^, ^49^, ^52^, ^53^] *N. gonorrhoeae* was grown on Chocolate Columbia agar plates at 37 °C+5 % CO_2_ and in proteose peptone medium supplemented with HEPES at 37 °C.

### SEEL of bacteria

One‐step SEEL was performed on bacteria grown in liquid culture (5×10^8^ bacteria). The bacteria were washed with buffer and were incubated with SEEL label mix at 37 °C for 2 h while rotating. A typical SEEL label mix (50 μL) was prepared in medium (PBS buffer pH=7.2 /HEPES buffer pH=7.2) with ST6Gal1 (1.05 μL of stock 1 mg/mL), CMP‐Sialic acid derivative (50 μM final concentration), 0.34 μL BSA (2 mg/mL stock concentration) and 0.34 μL alkaline phosphatase (1 U/μL stock concentration). After SEEL treatment, the bacteria were washed with buffer and prepared for application. The bacteria were typically pelleted at 4.0 krpm 1500 g for 5 min with a tabletop centrifuge.

Two‐step SEEL was performed similar to one‐step SEEL, followed by a click reaction. In case of CuAAC, 100 μL reaction volume contained 100 μM acetylene‐PEG_4_‐biotin, 500 μM CuSO_4_ and 2.5 mM sodium l‐ascorbate. In case of SPAAC 100 μL reaction volume contained 100 μM DBCO‐PEG_4_‐biotin. For the fluorophores AF488‐alkyne and DBCO‐AF488 the final concentration was 10 μM.

If the bacteria were heat‐inactivated, they were heated at 80 °C for 15 min and treated with the described SEEL method. As a result of heat‐inactivation, the bacteria are no longer viable.

### Western blotting

In case of (glyco)proteins, the samples were lysed and analyzed with a 10 % SDS‐PAGE gel for which the gel was run for 45–60 min at 150 V. For the western blotting, the gel was electroblotted onto a PVDF membrane. The membrane was blocked (5 % milk, 30–60 min), washed (1 % milk, 5 min), stained with anti‐biotin‐HRP antibody (1 : 20000 in 1 % milk), washed (1 % milk, followed by PBS, 5 min each), and treated with ECL western substrate for signal detection.

### LOS preparation and Tris‐Tricine gel

Samples were boiled for 5 min and then treated with protease K (10 μL, 20 mg/mL) overnight at 55 °C. Laemmli buffer (3×) was added and the samples were analyzed with a 16 % Tris‐Tricine gel. The gel ran typically for 3–4 h at 20 mA and was then further analyzed by Western blotting or silver staining or in‐gel fluorescence. Separate gels were used per analysis, because the methods for development are incompatible.

### Silver staining

Silver staining was performed on a 16 % Tris‐Tricine gel as described previously.^[67]^ Briefly, the gel was fixed (30 min, 40 % ethanol, 5 % acetic acid), oxidized (5 min, 0.7 % sodium periodic acid, 40 % ethanol, 5 % acetic acid), washed (3×5 min in distilled water), stained (distilled water containing 19 % 0.1 M NaOH, 1.3 %>28 % ammonium hydroxide, 3,3 % 20 % w/v silver nitrate), washed (3×5 min in distilled water), developed until bands appeared (distilled water containing 0.1 % PFA 37 % and 0.1 % citric acid 100 mg/mL), rinsed with distilled water and stopped (7 % acetic acid in distilled water).

### In‐gel fluorescence

In‐gel fluorescence was measured on Amersham imager 600 using the Green channel (520 nm, cy3).

### Flow cytometry

Samples were fixed with 1 % paraformaldehyde and 0.5 % BSA. If necessary, bacteria were diluted in PBS+0.05 % BSA to not exceed 20000 counts/s in flow cytometry analysis. The bacteria were gated as depicted in Supporting Figure S3A. Quantum beads of Alexa Fluro 488 MESF (Bangs Laboratories) were used. Flow cytometry was performed on MACSQuant flow cytometer (Miltenyi Biotech) and analysis was done with FlowJo Software (V10).

### Imaging

SEEL labeled bacteria (circa 3×10^8^) were centrifuged, resuspended in HEPES+1 % BSA and DAPI (4′,6‐diamidino‐2‐phenylindole) (1 : 50) was added. The samples were incubated for 25 min in the dark before washing (milliQ+0.1 % Tween) and then carefully resuspended in ProLong Diamond Antifade Mountant (P36961). Sample (5 μL) was taken, put on a poly‐l‐lysine coated coverslip and mounted on a glass slide. Slides were stored at RT overnight to allow the samples to harden and subsequently stored at 4 °C. Z stack images (25 steps of 0.23 μm) were recorded on a Yokogawa W1 spinning disk system (Evident SpinSR10 equipped with cellSense Dimension 3.2) using a 100× oil objective (UPLXAPO, NA1.45) with an ORCA fusion sCMOS camera (Hamamatsu) resulting in a pixel size of 65 nm. Sequential laser excitation of 405 and 488 nm recorded fluorescence emission using 477/60 (DAPI, exposed 200 ms) and 525/50 nm (FITC/SEEL, exposed 200 ms), respectively. The main dichroic mirror was a quadband (D 405/488/561/640 nm). The recordings were processed using OlyVIA software.

### MOE with Neu5Az

Neu5Az was synthesized according to a published procedure.^[68]^ MOE was performed with 6×10^8^ bacteria and these were incubated with indicated concentrations of Neu5Az in SI Figure S8. Bacteria were incubated for 6 h shaking at 160 rpm at 37 °C. After incubation, the samples were washed 2×1 mL (PBS buffer pH=7.2 or HEPES buffer pH=7.2) and clicked via CuAAC, see also SEEL of bacteria for the click conditions, were washed again and further treated according to LOS preparation.

### Growth measurements

Bacteria were treated according to conditions specified in text. Bacteria were diluted to OD=0.05 and the growth was monitored with Synergy HTX multi‐mode meter in a hypoxic glove box for 24 h while shaking continuously. Data was exported and analyzed with excel/prism.

### General Methods and Materials for the synthesis of CMP‐Sia‐AF488

Alexa Fluor 488 succinimidyl (NHS) ester was purchased from Thermo Fisher Scientific. Other reagents were obtained from commercial sources and used as purchased. Dichloromethane (DCM) was freshly distilled using standard procedures. Other organic solvents were purchased anhydrous and used without further purification. Unless otherwise noted, all reactions were carried out at room temperature (RT) in glassware with magnetic stirring. Organic solutions were concentrated under reduced pressure with bath temperatures <30 °C. Flash column chromatography was carried out on silica gel G60 (Silicycle, 60–200 μm, 60 Å). Thin‐layer chromatography (TLC) was carried out on Silica gel 60 F254 (EMD Chemicals Inc.) with detection by UV absorption (254 nm) where applicable, by spraying with 20 % sulfuric acid in ethanol followed by charring at ∼150 °C or by spraying with a solution of (NH_4_)_6_Mo_7_O_24_⋅H_2_O (25 g/L) in 10 % sulfuric acid in ethanol followed by charring at ∼150 °C. ^1^H NMR spectra were recorded on a Varian Inova 500 (500 MHz) spectrometer equipped with sun workstations or on a Bruker Ultrashield (600 MHz). Mass spectra were recorded on an Applied Biosystems 5800 MALDI‐TOF or Shimadzu LCMS‐IT‐TOF mass spectrometer. The matrix used was 2,5‐dihydroxy‐benzoic acid (DHB).

### Synthesis of Compound 3

To a solution of Alexa Fluor 488 NHS (1 eq, 1 mg) in DMF (680 μL), alkyne 2^[69]^ (3 eq, 0.44 mg) and triethylamine (9 eq, 970 μL) were added. The resulting mixture was stirred at room temperature for 30 minutes in darkness until ESI‐MS indicated completion of the reaction. After removing solvent under reduced pressure, the crude residue was concentrated to afford 1.1 mg of product as an orange solid in quantitative yield. The product was used in the next step without further purification. ESI‐MS m/z calcd for C_30_H_29_N_3_O_13_S_2_, [M‐1H]^−^: 702.1069, found, 702.1099.

### Synthesis of Compound 1 (CMP‐Sia‐AF488)

To a solution of compound **3** (1.3 eq, 1.1 mg) and CMP‐NeuAz^[38]^ (compound **4**, 1 eq, 0.79 mg) in 200 μL 0.1 M NH_4_HCO_3_ was added 0.1 M sodium l‐ascorbate (12.1 μL), 0.1 M CuSO_4_ (9.61 μL), and 130 μg TBTA. The resulting mixture was stirred at room temperature for 2 h 30 min in darkness. After completion of the reaction as indicated by ESI‐MS, the mixture was lyophilized. The residue was purified by a C18 column using a gradient of water and methanol (from 90/10 to 30/70, v/v) to afford compound **1** (1 mg, 62 %) as an orange solid. ^1^H NMR (600 MHz, D_2_O) δ=8.13–7.98 (m, 3H), 7.25–7.22 (m, 1H), 6.96–6.94 (m, 1H), 6.16–6.13 (m, 3H), 6.03–6.00 (m, 3H), 5.36–5.30 (m, 2H), 4.55–3.48 (m, 26H), 2.53–2.51 (m, 1H), 1.68–1.66 (m, 1H). See supporting Information Figure S12 for copies of the ^1^H‐NMR and TOCSY spectrum. ESI‐MS m/z calcd for C_50_H_60_N_10_O_29_PS_2_, [M‐1H]^−^: 1359.2712, found, 1359.2730.

## Abbreviations

Kdo: 3‐Deoxy‐D‐manno‐oct‐2‐ulosonic acid
NAM: *N*‐acetyl muramic acid
SEEL: selective exoenzymatic labeling
MOE: metabolic oligosaccharide engineering

LacNAc: *N*‐acetyllactosamine
LOS: lipooligosaccharides
CuAAC: copper‐catalyzed azide‐alkyne cycloaddition
DBCO: dibenzocyclooctyne
DAPI: 4′,6‐diamidino‐2‐phenylindole

## Author contributions

H.J.: Methodology, Validation and Formal Analysis. H.J. and J.E.M.: Investigation. M.J.M., G.J.B., J.Y.O., and B.W.B.: Resources. H.J., M.W., and T.W.: Conceptualization, Formal analysis. H.J., G.J.B, M.W., and T.W.: Writing – Original Draft. All authors: Writing – Review & Editing. M.W. and T.W.: Supervision. T.W.: Funding acquisition and Project administration.

## Conflict of interest

The authors declare no conflict of interest.

1

## Supporting information

As a service to our authors and readers, this journal provides supporting information supplied by the authors. Such materials are peer reviewed and may be re‐organized for online delivery, but are not copy‐edited or typeset. Technical support issues arising from supporting information (other than missing files) should be addressed to the authors.

Supporting Information

## Data Availability

All data supporting the findings of this study are available within the paper and within the supplementary data published online. Raw data are available from the corresponding author, (Tom Wennekes), upon request.
