# Supplementary material for: Selective Exoenzymatic Labeling of Lipooligosaccharides of Neisseria gonorrhoeae with α2,6‐Sialoside Analogues
Source: Chembiochem. 2022 Aug 23;23(19):e202200340. doi: 10.1002/cbic.202200340 (PMC9804176; doi:10.1002/cbic.202200340)
Supplement: Supplementary file 1 — Supporting Information [file CBIC-23-0-s001.pdf]

ChemBioChem

Supporting Information

Selective Exoenzymatic Labeling of Lipooligosaccharides of *Neisseria gonorrhoeae* with α2,6-Sialoside Analogues

Hanna de Jong, Maria J. Moure, Jet E. M. Hartman, Gerlof P. Bosman, Jun Yang Ong, Bart W. Bardoel, Geert-Jan Boons, Marc M. S. M. Wösten,* and Tom Wennekes*

Contents

Supplementary figure 1. 2
Supplementary figure 2.. 2
Supplementary figure 3. 2
Supplementary figure 4. 3
Supplementary figure 5. 3
Supplementary figure 6. 4
Supplementary figure 7. 5
Supplementary figure 8. 5
Supplementary figure 9.. 6
Supplementary figure 10. 7
Supplementary figure 11.. 8
Supplementary figure 12 9
Supplementary figure 13. 10

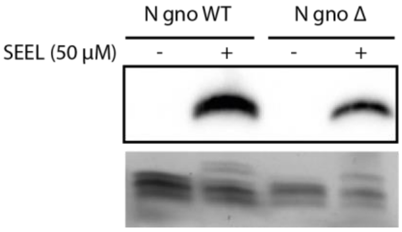

Supplementary figure 1. Western blot (upper panel) and silver stain (bottom panel) of LOS from one-step SEEL treated *N. gonorrhoeae* wildtype (WT) and mutant (Δ). These bacteria were not heat-treated before labeling by SEEL and the sialyltransferases present for wildtype contribute to the labeling of the LOS, as observed by the slightly stronger biotin signal for the wildtype compared to the sialyltransferase mutant bacteria. To exclusively study labeling by SEEL, which can introduce a non-native glycosidic linkage, either bacteria were heat treated or mutant bacteria were used.

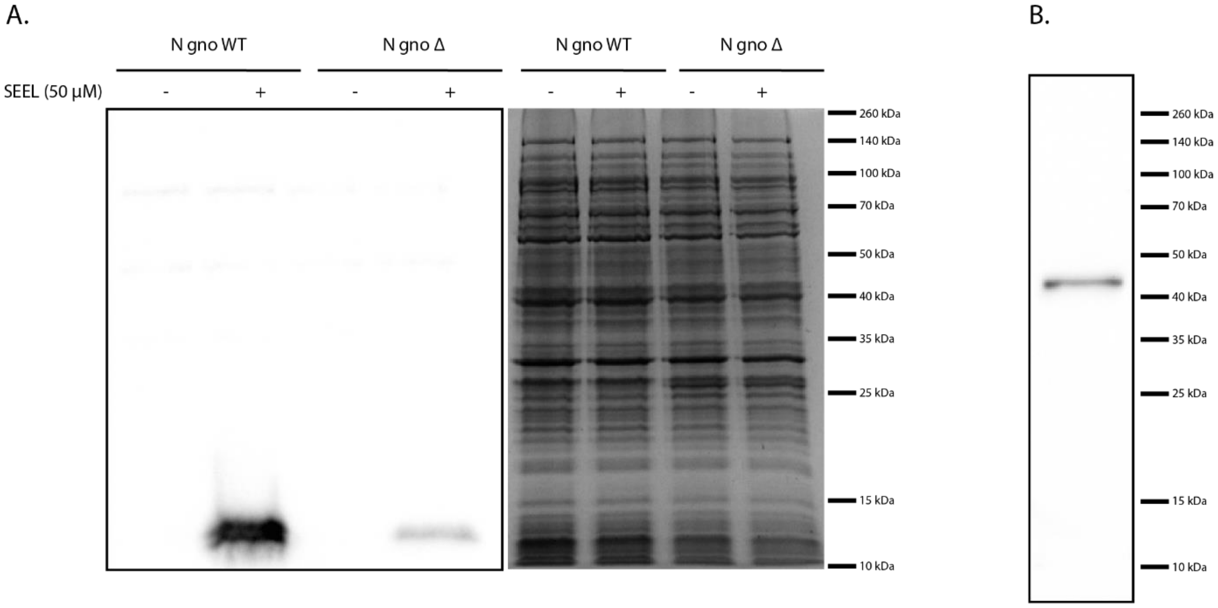

Supplementary figure 2. (A) Western blot and pageblue analysis of protein samples from SEEL treated *N. gonorrhoeae*. The bottom bands are labeled LOS. (B) SEEL label mix analyzed on Western blot. The enzyme ST6Gal1 (47 kDa) labels itself in solution.

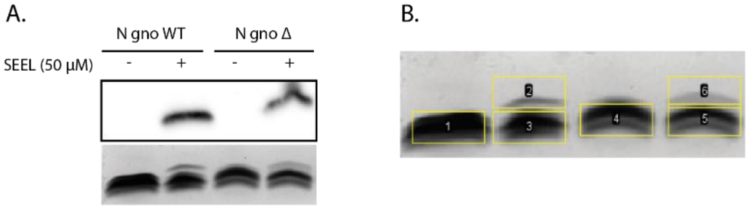

C.

Image area	Condition	Mean
1	N gno WT ctrl	43.567
2	N gno WT SEEL	182.983
3	N gno WT SEEL unlabeled	85.664
4	N gno delta ctrl	74.103
5	N gno delta SEEL unlabeled	89.784
6	N gno delta SEEL	177.518

Supplementary figure 3. (A) One-step SEEL after heat-inactivating *N. gonorrhoeae* wildtype (N gno WT) and mutant (N gno Δ). (B and C) The mean grey intensity of the bands in the silver stain were determined per fixed area via the image processing software ImageJ. From this image processing it was determined that roughly 32 % and 34 % of the LOS was labeled by SEEL for the wildtype and mutant, respectively. For the calculation: 100 – ((SEEL/ SEEL + SEEL unlabeled)*100) = % labeled by SEEL.

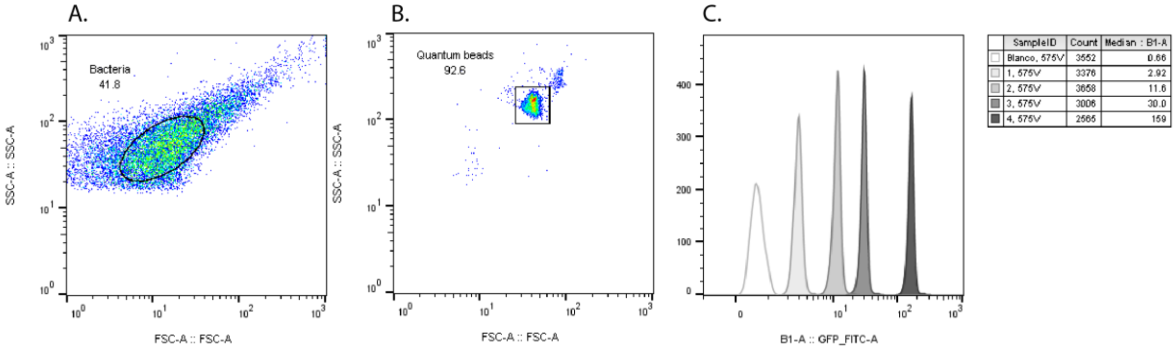

Supplementary figure 4. (A) Gating of *N. gonorrhoeae* for flow cytometry. (B) Gating of quantum beads for quantification of the number of fluorescence labels. (C) The amount of fluorescence in the 488 channel (FITC) for the different quantum beads with the median indicated in the legend. The conversion of median fluorescence intensity to Molecules of Equivalent Soluble Fluorochrome (MESF) was based on the calculations by the supplier of the quantum beads (Bangs laboratories, Quantum™ Alexa Fluor® 488 LOT 14506, Quickcal v2.3).

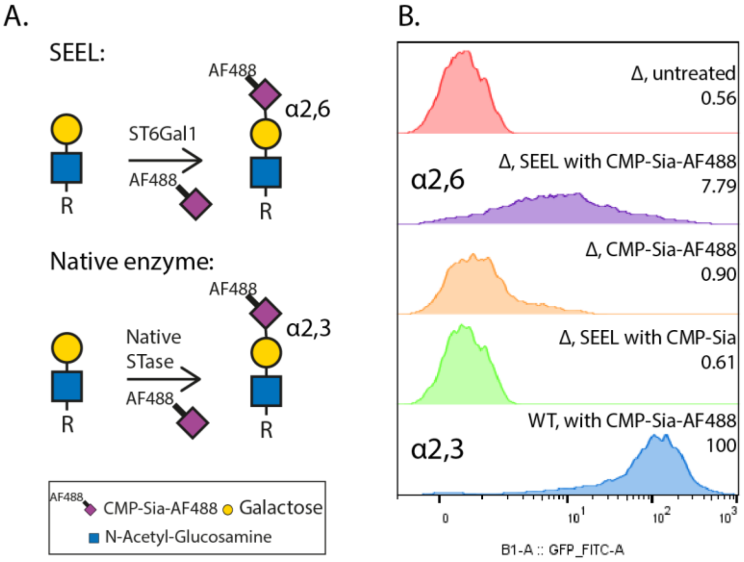

C.

	Average median of fluorescence intensity	Nr. of modifications (MESF for QBs)	Glycosidic linkage
**N gno Δ SEEL ±STdev**	5.9 ±3.2	19004 ±11647	AF488 α2,6
**N gno WT ±STdev**	69.0 ±27	352764 ±165398	AF488 α2,3

Supplementary figure 5. (A) Schematic overview of the LOS labeling for SEEL treated mutant bacteria and WT treated with nucleotide sugar only. (B and C) The level of modifications is higher in presence of native sialyltransferases than labeling by SEEL, yet a different glycosidic linkage is obtained through these approaches due to the inherent activity of the enzymes.

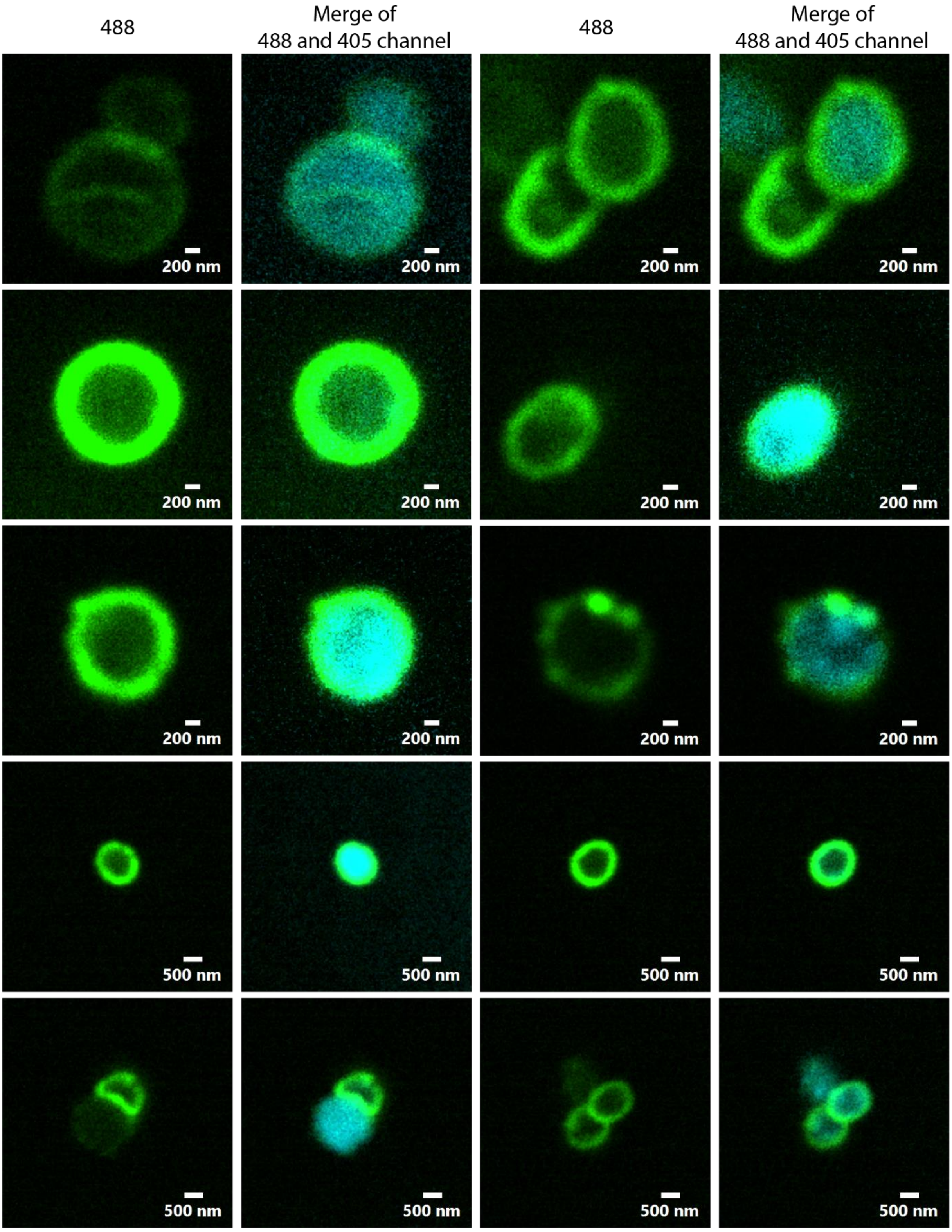

Supplementary figure 6. Fluorescence microscopy images of SEEL treated *N. gonorrhoeae* with CMP-Sia-AF488.

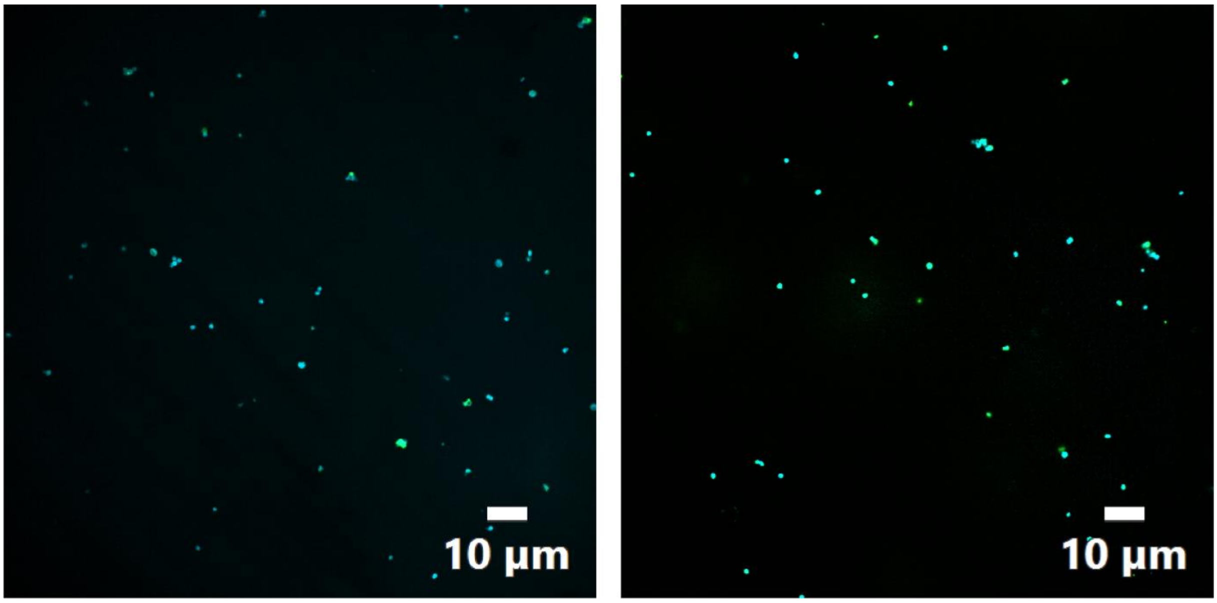

Supplementary figure 7. Fluorescence microscopy images of SEEL treated *N. gonorrhoeae* in two overviews (merge of 488 and 405 channel).

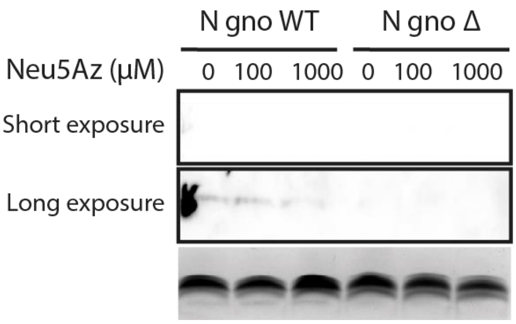

Supplementary figure 8. Metabolic oligosaccharide engineering of *N. gonorrhoeae* WT (N gno WT) and a sialyltransferase mutant (N gno Δ) with 5-azidoneuraminic acid (Neu5Az) at indicated concentrations does not show incorporation of the monosaccharide probe into the LOS.

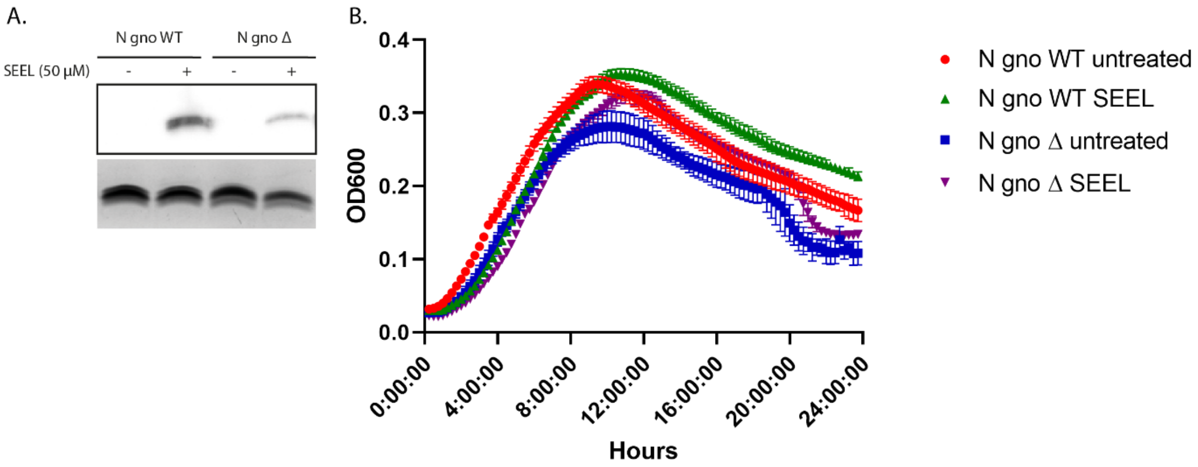

Supplementary figure 9. (A) One-step SEEL labeling in HEPES buffer, which is used for the cytotoxicity assays. (B) OD_{600} measured over time to test cell viability of untreated and SEEL treated bacteria for both wildtype and mutant strain of *N. gonorrhoeae*. One-step SEEL labeling with CMP-Sia-biotin of bacteria was performed according to the description in the Material and methods. After washing, the bacteria were prepared and measured as described in the section Growth measurements. Time point 0 h starts after the SEEL labeling and washing. The OD_{600} is reported as an average of a triplicate at 15 min intervals for 24 h.

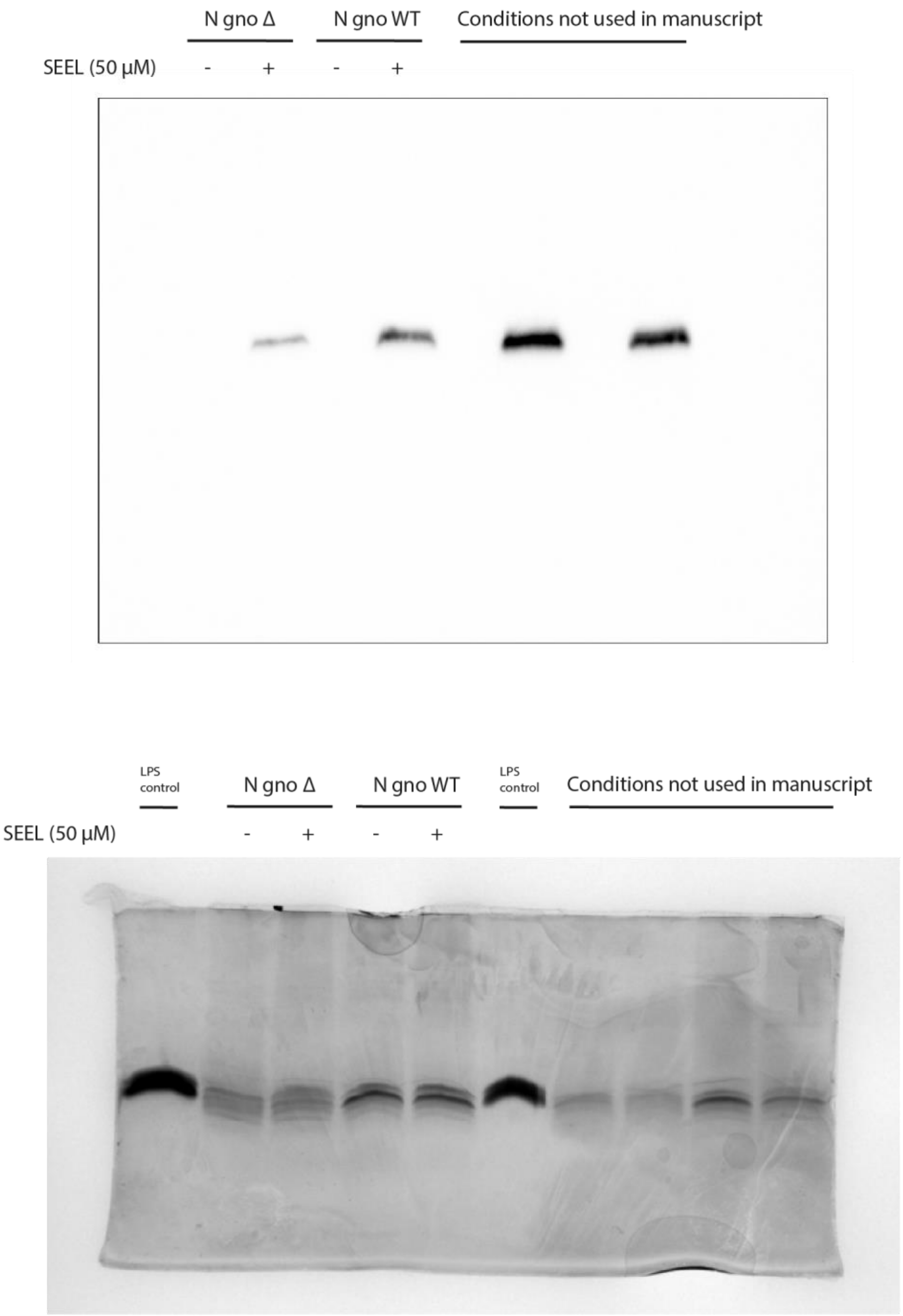

Supplementary figure 10. Raw data images of Western blot (top) and silver stain (bottom) that were used in Figure 2D.

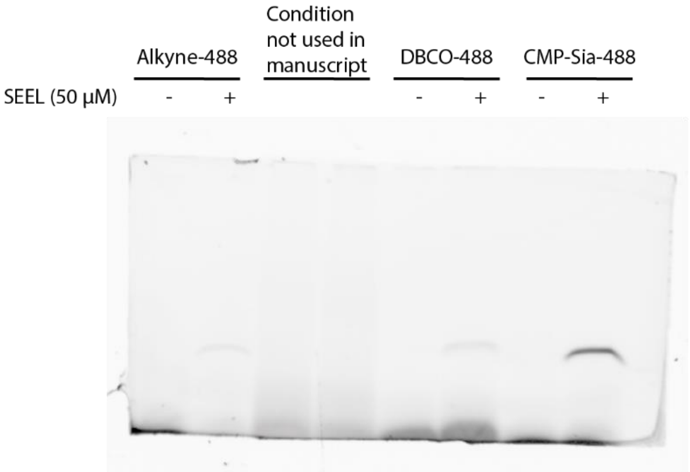

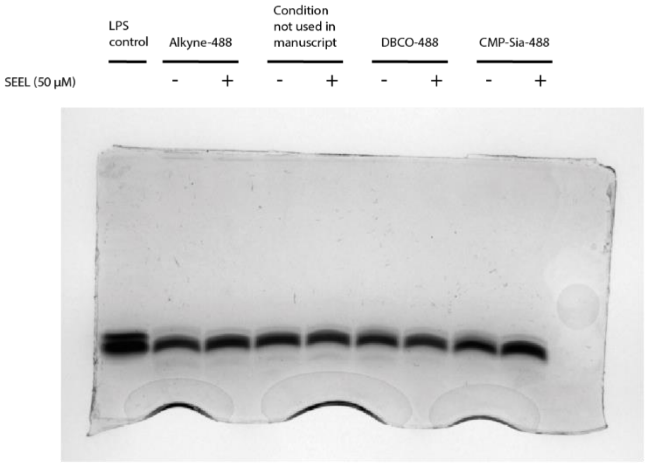

Supplementary figure 11. Raw data images of in-gel fluorescence (top) and silver stain (bottom) that were used in Figure 2E.

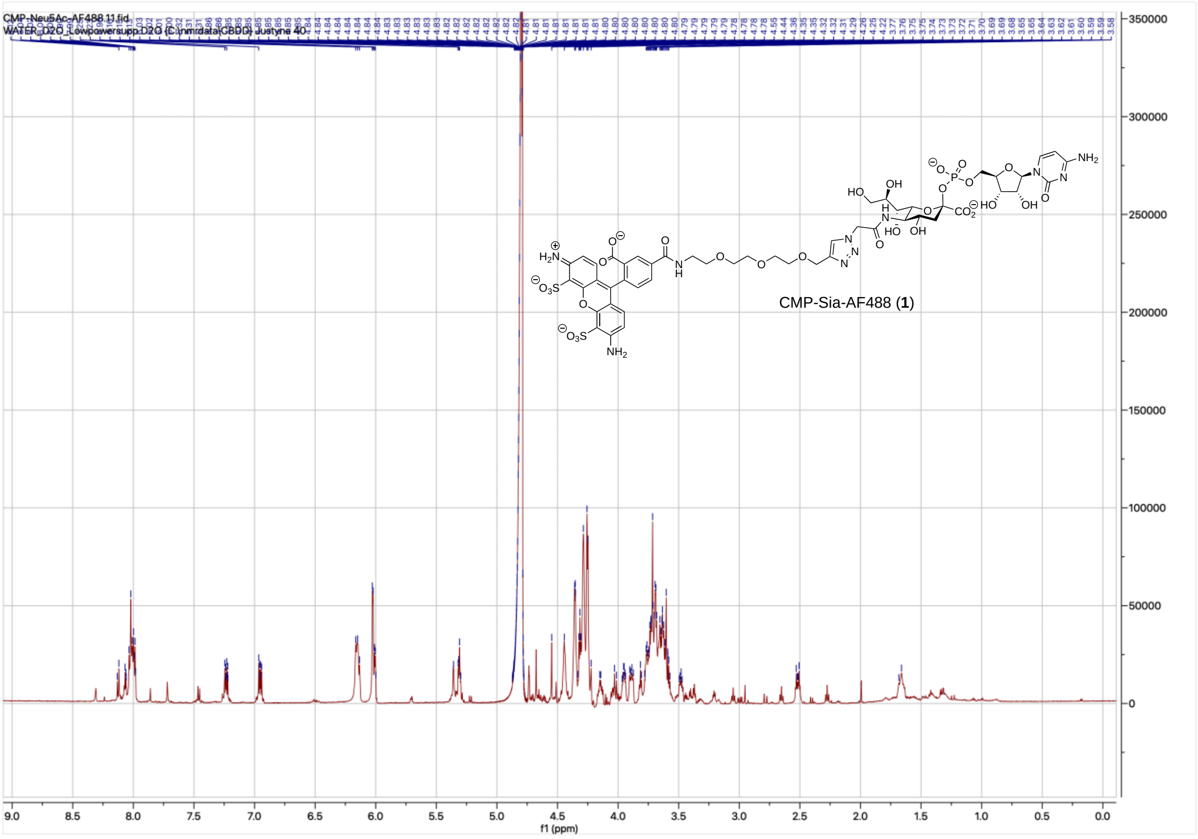

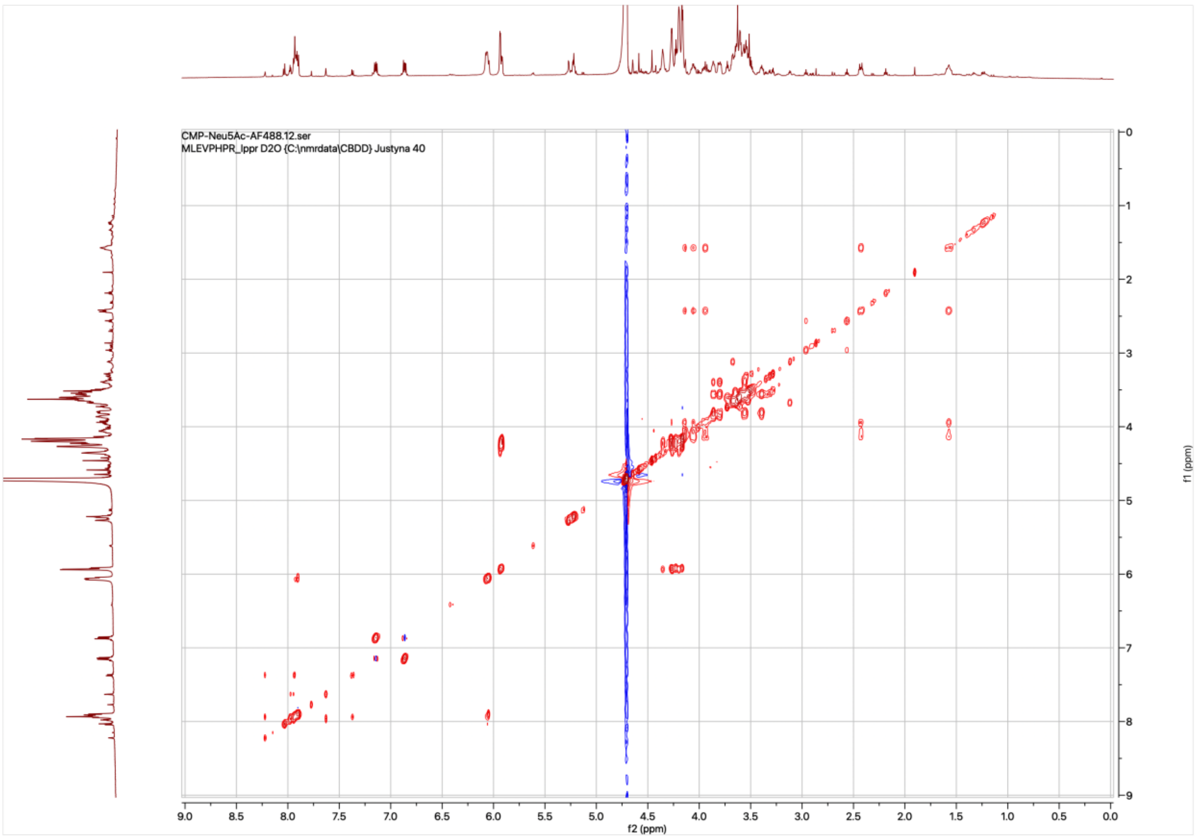

Supplementary figure 12 Copy of ^{1}H-NMR (top) and TOCSY (bottom) spectra for CMP-Sia-AF488 (**1**).

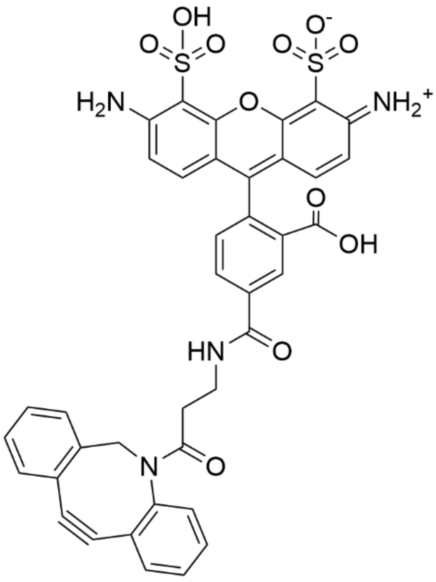

DBCO-AF488
Cat. no. supplier: CLK-1278

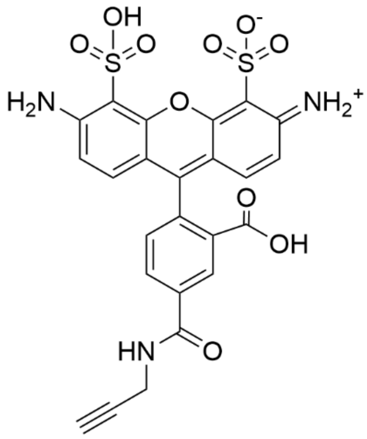

AF488-alkyne
Cat. no. supplier: CLK-1277

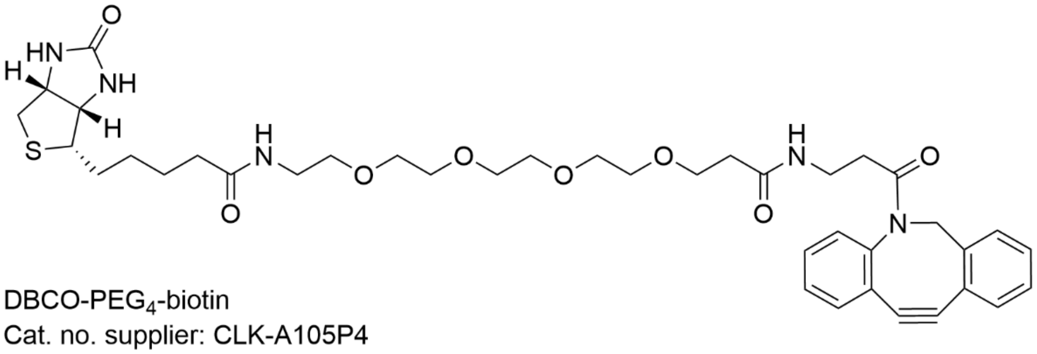

DBCO-PEG$_4$-biotin
Cat. no. supplier: CLK-A105P4

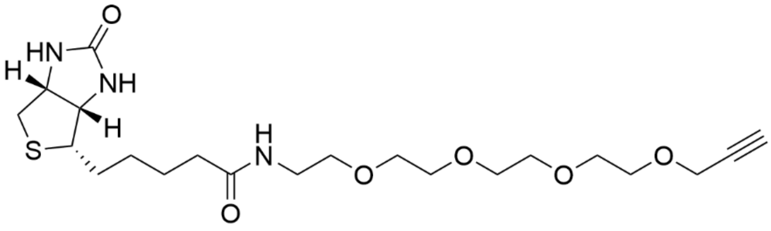

Acetylene-PEG$_4$-biotin
Cat. no. supplier: CLK-TA105

Supplementary figure 13. Chemical structures and catalog number by the supplier of the reagents used in click reaction in this manuscript.
